# Typewriter tinnitus revisited: The typical symptoms and the initial response to carbamazepine are the most reliable diagnostic clues

**DOI:** 10.1038/s41598-017-10798-w

**Published:** 2017-09-06

**Authors:** Woongsang Sunwoo, Yung Jin Jeon, Yun Jung Bae, Jeong Hun Jang, Ja-Won Koo, Jae-Jin Song

**Affiliations:** 1 Department of Otorhinolaryngology-Head and Neck Surgery, Gachon University Gil Medical Center, Incheon, Korea; 2 Department of Otorhinolaryngology-Head and Neck Surgery, Seoul National University Bundang Hospital, Seongnam, Korea; 3 Department of Radiology, Seoul National University Bundang Hospital, Seongnam, Korea; 4 Department of Otolaryngology-Head and Neck Surgery, Ajou University Hospital, Suwon, Korea

## Abstract

Although neurovascular compression of the cochlear nerve (NVC-C) presenting as typewriter tinnitus is a discrete disease category, verified diagnostic criteria are lacking. We sought to refine the diagnostic criteria for NVC-C by reference to a relatively large case series. The medical records of 22 NVC-C patients were retrospectively reviewed. Psychoacoustic characteristics, the results of diagnostic work-up (including audiovestibular neurophysiological tests and radiological evaluations), and the initial treatment response to carbamazepine were investigated. All subjects described their tinnitus as a typical “typewriter” or “staccato” sound. Of the 22 subjects, 11 (50%) had histories of vertiginous spells, but none had ipsilesional hearing loss. Vestibular function tests in 11 subjects tested revealed only 2 (18.2%) isolated cervical vestibular evoked myogenic potential abnormalities. Radiological comparisons of the symptomatic and asymptomatic sides, regarding the type of the vascular loop and neurovascular contact, revealed no significant differences. However, all 22 subjects exhibited immediate and marked responses to short-term carbamazepine treatment. Meticulous history-taking in terms of the psychoacoustic characteristics and the response to initial carbamazepine, are more reliable diagnostic clues than are radiological or neurophysiological data in NVC-C subjects. Therefore, the typical psychoacoustic characteristics and the response to initial carbamazepine should be included in the diagnostic criteria.

## Introduction

An artery compressing a cranial nerve can stimulate that nerve, triggering hyperactive cranial nerve syndrome with or without loss of function. Examples of hyperactive disorders associated with microvascular compression include trigeminal neuralgia (the fifth cranial nerve), hemifacial spasms (the seventh nerve), and glossopharyngeal neuralgia (the ninth nerve).

In the time since Mardini first reported “ear-clicking tinnitus responding to carbamazepine”, the idea that neurovascular compression of the cochlear nerve (NVC-C) by the loop of the anterior-inferior cerebellar artery (AICA) is responsible for such typewriter tinnitus has become accepted^1^. Typewriter tinnitus is characterized by unilateral staccato tinnitus, described using the adjectives “typewriter”, “Morse code”, or “machine-gun”, and paroxysmal attacks with intermittent tinnitus-free intervals. While non-pulsatile subjective tinnitus is presume to be resulting from secondary functional changes in auditory or non-auditory brain areas^2–8^ and responds very poorly to almost all kinds of medical treatment, typewriter tinnitus responds very well to carbamazepine^9–13^.

Paroxysmal vertigo, also termed disabling positional vertigo, is assumed to be caused by compression of the vestibular part of the eighth cranial nerve by the loop of the AICA. Several terms have been coined to describe a group of audiovestibular symptoms including cochleovestibular nerve compression syndrome^14^ and cochleovestibular compression syndrome^15^. However, although NVC-C has long been regarded as a discrete disease category attributable to vascular compression of the eighth nerve, few verified diagnostic criteria are available. Furthermore, most previous studies focused on paroxysmal vertigo^16–18^; only a few case reports have explored typewriter tinnitus^9, 12^.

Thus, we evaluated the clinical characteristics, data from diagnostic work-ups (including audiovestibular neurophysiological tests and radiological evaluations), and the initial treatment responses in a relatively large group of patients with typewriter tinnitus. We propose refined diagnostic criteria for patients with NVC-C.

## Materials and Methods

### Subjects

The study protocol and a waiver of consent for retrospective chart review were approved by the institutional review board of the Clinical Research Institute at Seoul National Bundang Hospital (approval no. B-1608–360–101). All methods employed in this study were in accordance with the approved guidelines and the Declaration of Helsinki. Data were collected from an electronic medical records database. All personal information was kept confidential as required. All clinical data were retrospectively reviewed by two otologists. From January 2014 to November 2016, 28 patients were initially diagnosed with typewriter tinnitus at our tinnitus clinic. Detailed histories of tinnitus were obtained; the time of onset, the psychoacoustic nature of tinnitus, the affected side(s), and associated symptoms (e.g., paroxysmal vertigo or hemifacial spasms). All subjects visited the outpatient clinic of the department of otorhinolaryngology with typical symptoms of typewriter tinnitus (i.e., paroxysmal “typewriter”, “machine-gun”, or “crackling” sounds). Of the 28 patients, 1 with a cerebral infarction and 5 lost to follow-up were excluded from the study; thus, 22 subjects (5 males, 17 females) of mean age 51.2 ± 15.8 years (range, 23–84 years) were finally included. No patient had any history of neurological disease, a tumor in the internal acoustic canal (IAC) or cerebello-pontine angle (CPA), or temporal bone trauma.

### Audiovestibular neurophysiological tests

The otoscopic findings and the pure tone audiometry (PTA) data of all patients were evaluated. The mean hearing level (MHL) was calculated as the average hearing threshold at 0.5, 1, 2, and 3 kHz^19^. Normal hearing was defined as an MHL ≤ the 25-decibel hearing level (dB HL).

For all patients presenting with typewriter tinnitus, vestibular function tests (VFTs), including the bithermal caloric test; recording of ocular vestibular evoked myogenic potentials (oVEMPs) and cervical VEMPs (cVEMPs); and the rotational chair test, were recommended at the initial visit. Of the 21 patients, 10 underwent at least one of these VFTs. Vestibular function was considered abnormal when: (1) canal paresis (CP, %; calculated using Jongkee’s formula) was > 25%^20^; or (2) when the VEMPs were reduced or absent on the affected side (oVEMP or cVEMP asymmetry ratio >40% or >33%, respectively^21^); or (3) when the slow harmonic acceleration (SHA) phase of the rotational chair test exhibited a reduced gain or an increased phase lead, in at least three consecutive frequencies of those tested (0.01, 0.02, 0.04, 0.08, 0.16, 0.32, and 0.64 Hz), using the manufacturer-provided normative data as references (Neuro Kinetics, Inc., Pittsburgh, PA, USA)^22^.

### Radiological evaluation

**Figure 1:**
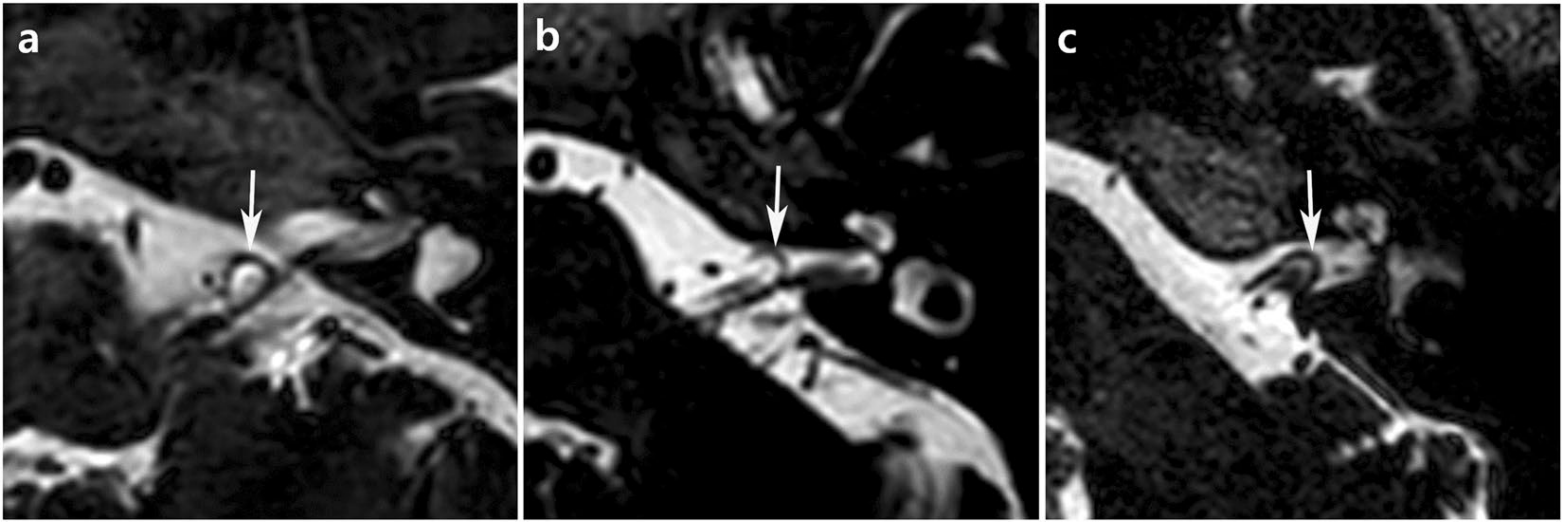
The types of AICA loops. (**a**) Type I loop is lying within the CPA (arrow) but not entering the IAC. (**b**) Type II loop is entering the IAC (arrow) within <50% of the length of the IAC. (**c**) Type III loop is extending into more than 50% of the IAC (arrow).

**Figure 2:**
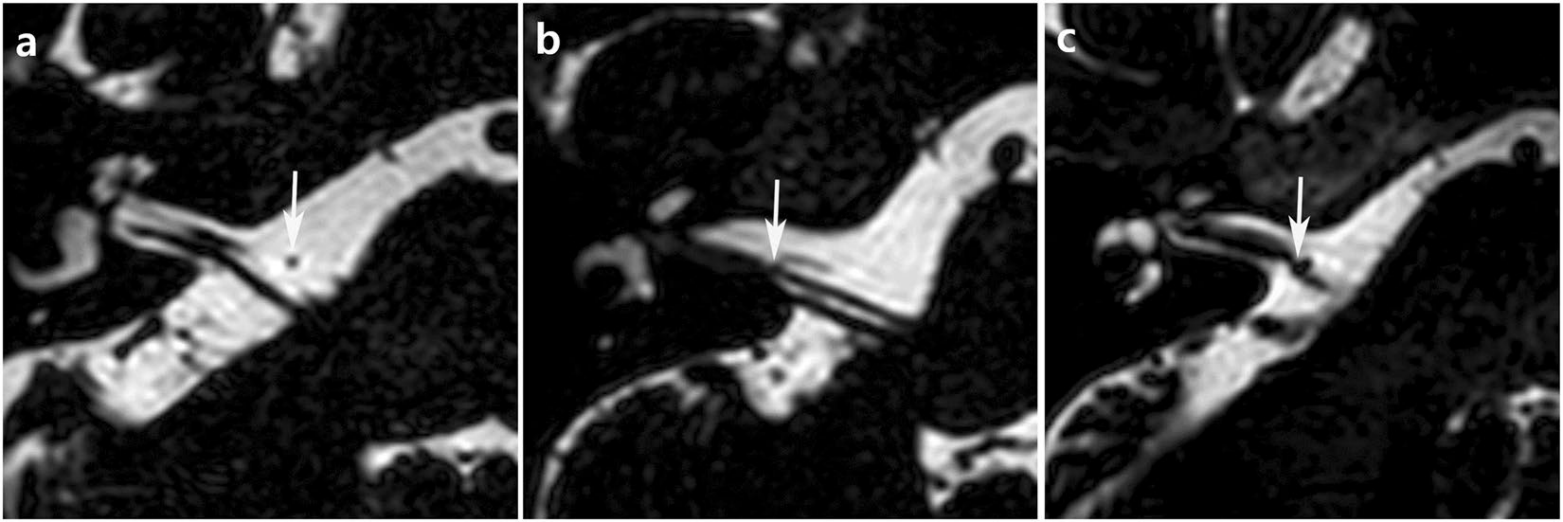
The types of neurovascular contact. (**a**) There is no neurovascular contact (arrow), which is classified as type I. (**b**) Type II shows neurovascular contact (arrow) between AICA and cochleovestibular nerve without angulation/indentation of the nerve. (**c**) Arrow indicates the angulation/indentation of cochleovestibular nerve by AICA loop in type III.

All subjects presenting with typewriter tinnitus were recommended to undergo magnetic resonance imaging of the internal acoustic canal (IAC-MRI). Of the 21 subjects, 14 actually underwent this. Two neuro-radiologists blinded to the clinical findings separately evaluated the neurovascular features of the IAC and the cerebello-pontine angle (CPA) on axial, three-dimensional (3D), T2-weighted, volume, isotropic, turbo, spin-echo acquisition (T2-VISTA) sequences obtained via 3T-MRI, and made final decisions by consensus. The types of AICA loops were defined using the Chavda classification: Type I, the AICA loop lay within the CPA but did not enter the IAC; Type II, the AICA loop lay within <50% of the length of the IAC; and, Type III, the AICA loop extended into >50% of the IAC (Fig. 1)^23^. In addition, the types of neurovascular contact were classified into the following categories using the system of Gultekin *et al*.: Type I, no contact; Type II, vascular contact without angulation of the cochleovestibular nerve; and Type III, angulation of the cochleovestibular nerve caused by neurovascular compression (Fig. 2)^24^.

### Response to initial treatment with carbamazepine

After excluding other possible pathological causes of the tinnitus by careful history-taking and otological/neurological examinations, all patients were prescribed carbamazepine as an initial empirical treatment. Oral carbamazepine was initiated at 200–400 mg/day by reference to body weight. When a definite response to carbamazepine was evident, the daily dose was adjusted to the minimum effective level (100 mg/day in most cases). Over a maximum period of 3 months, all patients were followed up by blood testing, including complete blood counts^25, 26^. No patient ceased treatment because of side effects such as leukopenia or thrombocytopenia. The responses to carbamazepine were divided into three categories: (1) no response (NR); (2) partial remission (PR) (marked or partial relief); and (3) complete remission (CR) (complete suppression of the tinnitus).

### Statistical analysis

Data are presented as means ± standard deviations. Fisher’s exact test was used to compare the VFT results by the presence of vertigo. The types of AICA loops and the types of neurovascular contacts evident on MRI were compared between the symptomatic and asymptomatic sides using the linear-by-linear association test. Statistical significance was set at *P* < 0.05. All statistical analyses were performed with the aid of SPSS software (version 18.0; SPSS, Inc., Chicago, IL, USA).

## Results

### Demographic characteristics and principal complaints

The mean duration of tinnitus was 14.6 months (range, 2 weeks to 10 years). In total, 19 of the 22 patients (86.4%) had tinnitus histories <2 years, whereas the other 3 had tinnitus for >3 years. The tinnitus was unilateral in 21 patients and bilateral in 1 (case no. 9, Table 1). Of the 21 patients with unilateral tinnitus, the right ear was affected in 10 and the left in 11.

**Table 1:** Raw Data for 22 Cases of Typewriter Tinnitus.

Case No.	Sex/Age	Side	Duration of history (months)	Vertigo	Hearing impairment, MHL (dB HL)	Facial spasm	Carbamazepine responsiveness	MRI findings		Vestibular dysfunction		
								AICA loop type^a^	Contact type^b^	Caloric, CP (%)	oVEMP/cVEMP	Rotational chair test
1	F/31	R	0.75	−	−, 3	−	+, CR	I	III	N/A	N/A	N/A
2	F/52	L	0.5	+	−, 16	−	+, PR	II	I	−, 4	−/+	−
3	M/47	L	0.75	+	−, 10	+	+, PR	I	III	N/A	N/A	N/A
4	F/43	R	7 years	+	+, 45	−	+, PR	I	II	−, 11	−/−	−
5	F/58	R	3	+	−/8	−	+, PR	II	II	−, 17	−/−	N/A
6	F/84	L	1	−	+, 45	−	+, PR	I	I	N/A	N/A	N/A
7	M/77	L	8	−	+, 53	+	+, PR	I	I	N/A	N/A	N/A
8	M/39	L	6	−	−, 3	−	+, PR	III	II	N/A	N/A	N/A
9	M/65	R/L	10 years	−	−, 21/+, 30	+	+, PR	II, II	II, I	N/A	N/A	N/A
10	F/72	L	5	+	+, 28	−	+, PR	I	II	−, 0	N/A	−
11	F/40	L	1 year	−	−, 5	−	+, PR	I	I	N/A	N/A	N/A
12	F/51	R	2	+	−, 8	−	+, PR	N/A	N/A	−, 3	N/A	−
13	F/56	R	2 years	−	−, 12	−	+, PR	N/A	N/A	−, 4	N/A	−
14	F/27	R	6	−	−, 10	−	+, PR	N/A	N/A	−, 4	−/+	−
15	F/56	L	4	+	−, 3	−	+, PR	I	II	−, 1	−/−	N/A
16	F/23	R	1	+	−, 0	+	+, PR	I	I	−, 16	−/−	−
17	F/66	R	2	+	−, 8	−	+, PR	N/A	N/A	N/A	N/A	N/A
18	M/45	R	6	+	−, 5	−	+, PR	N/A	N/A	N/A	−/−	N/A
19	F/34	L	N/A	−	−, 6	−	+, PR	N/A	N/A	N/A	N/A	N/A
20	F/49	R	1	−	−, 18	−	+, CR	N/A	N/A	N/A	N/A	N/A
21	F/54	L	1.5	+	−, 14	−	+, CR	II	III	N/A	N/A	N/A
22	F/58	L	1 year	−	−, 8	+	+, PR	I	II	−, 13	−/−	N/A

MHL, mean hearing level, the average hearing threshold at 0.5, 1, 2, and 3 kHz; AICA, anterior-inferior cerebellar artery; CP, canal paresis; CR, complete remission; PR, partial remission; N/A, not available.

^a^The type of AICA loop according to Chavda classification^23^.

^b^The type of neurovascular contact according to Gultekin and colleagues’ classification^24^.

Eight patients (36.4%) complained of typewriter tinnitus without any other neurotologic symptoms, while the other 14 (63.6%) had combined neurotologic symptoms. That is, 3 patients had combined ipsilateral hemifacial spasm, 9 had vertigo, and 2 complained of both hemifacial spasm and vertigo. Of 22 patients, 5 had bilateral symmetric mild to moderate sensorineural hearing loss.

### Hearing impairment

The MHL of all patients was 15.6 ± 14.9 dB HL on the affected side(s). However, in all five patients with SNHL (median: 45 dB HL, range: 28–53 dB HL), the differences in hearing thresholds at all frequencies were <15 dB HL between the ipsi- and contra-lesional sides, thus not meeting the criteria for asymmetric SNHL^27^. Of five patients with hearing impairment, one had typewriter tinnitus on both sides. In four patients with unilateral tinnitus, the MHL asymmetries between the two ears ranged from 1 to 8 dB HL.

### Vestibular dysfunction

Of the 11 patients who underwent vestibular function tests, 2 (18.2%) exhibited abnormal results. Of the two cases of vestibular dysfunction, one (case 2) presented with vertigo. Two patients (cases 2 and 14) exhibited only isolated abnormal cVEMP results; amplitudes were reduced in the affected ears and asymmetry ratio was 51.9% and 47.9%, respectively. We found no association between the presence of vertigo and abnormal results on vestibular dysfunction testing (*P* = 1.00, Fisher’s exact test).

### Response to carbamazepine

All 22 patients responded positively to carbamazepine: CR was achieved in 3 cases (13.6%) and PR in 19 (86.4%). No instance of NR was noted. All 11 patients with both typewriter tinnitus and paroxysmal vertigo exhibited improvements in both the tinnitus and the vertigo. However, when they stopped their medication for a few days, the symptoms recurred. Of five patients with hemifacial spasms, three (60%) reported improvements after carbamazepine treatment.

### MRI findings

Of the 22 patients, 15 underwent neuroimaging evaluation. Of 16 symptomatic sides (thus including one patient with bilateral typewriter tinnitus), we recorded 10 instances of type I AICA loops (62.5%), 5 of type II loops (31.3%), and 1 of a type III loop (6.3%). Of the 14 AICA loops of the asymptomatic sides, 9 were classified as type I (64.3%), 3 as type II (21.4%), and 2 as type III (14.3%). The anatomical locations of the AICA loops in the IAC and CPA did not differ significantly by the presence or absence of typewriter tinnitus (*P* = 1.00, linear-by-linear association).

Type III neurovascular contact (compression with nerve angulation/indentation) was evident in 3 of 16 sides (18.8%) with typewriter tinnitus. One of 14 (7.1%) sides without tinnitus exhibited type III neurovascular contact. Such contact (without nerve angulation/indentation) was evident on seven (43.8%) symptomatic sides and six (42.9%) asymptomatic sides. Six (37.5%) symptomatic sides and seven (50%) asymptomatic sides exhibited no neurovascular contact. The type of neurovascular contact did not differ significantly between the symptomatic and asymptomatic sides (*P* = 0.439, linear-by-linear association). In addition, no vascular structure other than the AICA loop contacted the cochleovestibular nerve.

## Discussion

We analyzed the clinical characteristics, the results of audiovestibular neurophysiological tests and radiologic evaluations, and the initial treatment response to carbamazepine, in a relatively large group of patients with typewriter tinnitus. All patients showed CR or marked responses to short-term carbamazepine treatment, but neither the audiovestibular nor the radiological examinations revealed consistent ipsilesional abnormalities at levels >50% except for the type of neurovascular contact (types II and III combined, 62.5%). In short, the most consistent findings were the response to carbamazepine, followed by abnormal ipsilesional radiological findings.

### The suggested pathophysiology of typewriter tinnitus and the possible role played bycarbamazepine

As mentioned above, an artery compressing a cranial nerve may stimulate that nerve triggering hyperactive cranial nerve syndromes [i.e., trigeminal neuralgia (the trigeminal nerve), hemifacial spasms (the facial nerve), vestibular paroxysmia (the vestibular nerve), and typewriter tinnitus (the cochlear nerve)]. The precise pathophysiological mechanism of neurovascular compression syndrome remains controversial. Although histopathological data are lacking for most cases of the syndrome, several possible pathophysiologies have been suggested by those studying various microvascular conflict disorders. For example, trigeminal neuralgia has been suggested to reflect neurovascular compression of the trigeminal nerve in the cistern of the cerebello-pontine angle^28, 29^; an inflammatory reaction of the nerve trunk may induce ectopic activity and potentially cause spontaneous pain^30^. In addition, in patients with hemifacial spasms, vascular compression of the facial nerve has been suggested to cause focal demyelination, reorganization, and axonal hyperactivity triggering the spasmodic symptoms^31, 32^. Schwaber *et al*. studied the histopathology of the vestibular nerve and suggested that the vascular loop did not play a primary role in the development of vestibular paroxysmia, but rather applied pressure to, or otherwise triggered, an ectopic excitation focus^33^. In fact, vascular looping around the internal auditory meatus does not always compress the eighth cranial nerve^34^, and thus some authors have emphasized that “conflict”, rather than “compression”, is the correct term^15^.

Carbamazepine (5H-dibenz[b,f]azepine-5-carboxamide) is an anticonvulsant widely used to treat partial and generalized epilepsy. The anticonvulsant effects of carbamazepine are attributable to partial inhibition of voltage-gated sodium channel activity. Therefore, carbamazepine reduces the frequency of the action potential by retarding recovery of the voltage-gated sodium channel after inactivation^35^.

Carbamazepine has also been used to treat various non-epileptic neurological disorders. In particular, it has been shown to reduce ectopic discharges from injured nerves (again by blocking the sodium channel) in patients with various neurovascular compression syndromes. For example, carbamazepine is one of the most extensively studied drugs for the treatment of trigeminal neuralgia. It has been evaluated in randomized clinical trials and is considered as the first-line treatment of choice for trigeminal neuralgia^25, 36, 37^. In addition, carbamazepine has occasionally been reported to relieve hemifacial spasms^38, 39^. It is also evidently effective in most patients with vestibular paroxysmia, even at low doses (200–600 mg/d)^40^. As is also true of other neurovascular compression syndromes, several anecdotal reports on small numbers of patients have claimed that carbamazepine is effective in patients with typewriter tinnitus^1, 9, 11, 12^. Similarly, in the present study on a relatively large number of such patients, short-term carbamazepine treatment caused the symptoms to disappear or markedly improve in all subjects. We suggest that, as is true of other neurovascular compression syndromes, carbamazepine-mediated inhibition of the voltage-gated sodium channel may suppress ephaptic axonal transmission in the injured cochlear nerve, and thus alleviate typewriter tinnitus.

### Revisiting the previous diagnostic criteria for NVC-C

Various criteria have been used to diagnose NVC-C; these include the characteristic psychoacoustic features of tinnitus and the associated symptoms. In addition to history-taking, Moller *et al*. developed neurophysiological criteria; auditory brainstem responses (ABR) were employed to diagnose NVC-C^41^. Radiological studies have also sought to define characteristic MRI findings of typewriter tinnitus^9, 12^.

In 2007, De Ridder *et al*. developed diagnostic criteria for cochleovestibular compression syndrome (CVCS), which are now widely accepted^15^. They are: (1) unilateral paroxysmal tinnitus; (2) co-existent ipsilateral symptoms including hemifacial spasms, otalgia, vertiginous spells, or hearing losses at tinnitus frequencies; (3) MRI findings evidencing vestibular conflict; and (4) abnormal ABR^15^.

The differential diagnosis of typewriter tinnitus includes short attacks of unilateral tinnitus. In addition to the previously suggested features of the condition (unilateral, paroxysmal, and short-lasting), we emphasized the “staccato” character of the complaint when evaluating the psychoacoustic characteristics. All subjects described their tinnitus as tapping, clicking, or crackling in nature. Although one patient presented with bilateral tinnitus, all other psychoacoustic characteristics were typical and the patient was thus included in our current case series. Although hemifacial spasms and trigeminal neuralgia can be diagnosed solely on the basis of unilateral paroxysmal characteristics including symptom-free intervals, the diagnosis of typewriter tinnitus is less straightforward. As tinnitus can present with various psychoacoustic characteristics, NVC-C presenting with typewriter tinnitus can only be diagnosed after all other possible causes of tinnitus have been ruled out. In a previous study on the surgical outcomes of microvascular decompression, less strict selection criteria were adopted and, as a result, >30% of cases did not respond to surgical treatment^34^. In this context, meticulous history-taking in terms of the psychoacoustic characteristics of the tinnitus is of utmost importance for patient evaluation and management planning.

Turning to the associated ipsilateral symptoms, 22.7% of our current patients had hemifacial spasms and 50% histories of vertiginous spells. As all five cases with hearing impairment presented with bilaterally symmetrical hearing thresholds, none of these patients seemed to have ipsilateral hearing loss driven only by NVC-C. In addition, the vestibular function tests yielded normal caloric or rotation chair data from all 11 subjects tested; only 2 (18.2%) exhibited isolated cVEMP abnormalities inadequate to allow diagnosis of ipsilateral vestibular dysfunction. As the facial, vestibular, and cochlear nerves lie close together in the internal auditory canal, NVC-C can trigger concomitant hemifacial spasms or vestibular paroxysmia. The presence of an accompanying neurovascular compression syndrome increases the possibility that NVC-C is the cause of tinnitus. However, we found that concomitant symptoms (other than paroxysmal vertigo) were rarely evident in subjects with NVC-C, in agreement with previous anecdotal findings in a small number of patients. Thus, none of the six patients studied by Levine and none of the four studied by Brantberg had any associated ipsilateral symptom^9, 12^.

In the current study, we evaluated the MRI data of 15 patients with typewriter tinnitus in terms of radiological evidence of NVC-C. We found that radiological findings suggestive of neurovascular compression of the cochlear nerve were not pathognomonic, but did aid in the diagnosis of typewriter tinnitus. Thus, according to the Chavda classification, 35.7% of asymptomatic sides had AICA loops that entered the IAC. Fifty percent of asymptomatic sides exhibited neurovascular contact with or without angulation/indentation. In previous studies, vascular loops in the CPA were frequently found in asymptomatic normal populations^42, 43^. Other studies found no significant association between tinnitus and any of the following variables: type of vascular loop, vascular contact, or angulation of the cochlear nerve^23, 24^. Thus, any role for MRI in the diagnosis of typewriter tinnitus may be more ancillary than salient. However, MRI is very useful to rule out other pathologies, such as lesions of the CPA and IAC.

Abnormal ABR, such as a reduced peak II amplitude and a prolongation of interpeak latency (IPL) between peaks I and III, have repeatedly been reported to be diagnostic for NVC-C^15, 41, 44^. Patients with short histories of the condition usually present with normal amplitudes and latencies, but, after 2 years of symptoms, the amplitude of peak II decreases^15, 34^. In the present study, 85.7% of patients had short histories of symptoms (≤1 year) and none of these patients exhibited hearing impairment. Thus, the ABR were not routinely evaluated.

### Response to carbamazepine may be the most reliable diagnostic clue in subjects with NVC-C

All 22 subjects improved markedly when on short-term carbamazepine treatment. In terms of the associated symptoms, paroxysmal vertigo and the hemifacial spasms abated in 100% and 60% of patients, respectively. As also found in previous case reports, all patients exhibited prompt symptom improvement, within 2 weeks of the commencement of low-dose carbamazepine^9, 12^. As prior studies revealed that carbamazepine did not benefit other tinnitus patients^45, 46^, the prompt response to low-dose carbamazepine may be the most reliable diagnostic criterion of typewriter tinnitus. In addition, considering that the previously suggested diagnostic criteria afforded a diagnostic accuracy of <50% in our current case series, some typewriter tinnitus patients can be diagnosed only by meticulous history-taking in terms of the psychoacoustic characteristics of their tinnitus, and a positive response to low-dose carbamazepine. Therefore, responsiveness to low-dose carbamazepine should be the first-line diagnostic criterion for typewriter tinnitus. We thus suggest a new diagnostic criteria for NVC-C that modifies previously suggested diagnostic criteria^15^ (Table 2).

**Table 2:** Modified diagnostic criteria for neurovascular compression of the cochlear nerve (NVC-C).

Consider diagnosis when paroxysmal, short-lasting tinnitus observed.
a. Staccato character (tapping, clicking, or crackling in nature)
b. Typically, unilateral, but bilateral involvement can also be seen.
1. Responsiveness to low-dose carbamazepine (200–400 mg/day)
a. Prompt tinnitus improvement (within 2 weeks)
b. When the medication is stopped, the symptoms may recur.
2. Associated ipsilateral symptoms
a. Paroxysmal vertiginous spells
b. Hemifacial spasms
3. Supportive criteria – positive auditory evoked potential using Moller’s criteria^41^
a. Commonly normal before 2 years of symptoms
b. Ipsilateral interpeak latency I-III ≥ 2.3 ms
c. Reduced peak II amplitude <33%
4. Exclusionary criteria for NVC-C
a. Tumor of the cerebellopontine angle or internal auditory canal on MRI
b. Demyelination disease on cerebral MRI
5. Positive MRI for vascular conflict have no diagnostic specificity.

### Limitations of the current study, and proposed future studies

To the best of our knowledge, this is the first study to show that meticulous history-taking and the response to initial carbamazepine are more reliable diagnostic clues than the previously suggested diagnostic criteria in many typewriter tinnitus patients. However, our current study had certain limitations. First, as we have already acknowledged, we could not evaluate changes in the latency and amplitude of the ABR; these were not evaluated in most patients because their symptoms were of short duration. Future studies exploring the diagnostic utility of the ABR in a larger number of subjects varying in symptom duration are needed to verify our results. Second, although carbamazepine is known to be ineffective for most forms of tinnitus (as mentioned above)^45, 46^, some studies have reported small effects of carbamazepine in patients with chronic tinnitus^47, 48^. Future studies comparing typewriter tinnitus to conventional pure-tone or narrow-band-noise-like tinnitus in terms of the response to carbamazepine are therefore required.

In conclusion, we suggest that meticulous history-taking in terms of subjective psychoacoustic characteristics, and the response to initial carbamazepine, are more reliable diagnostic clues than are radiological or neurophysiological findings in patients with typewriter tinnitus. In other words, radiological or neurophysiological abnormalities are of limited diagnostic utility. Therefore, the typical psychoacoustic characteristics and the response to initial carbamazepine should be included in the previously suggested diagnostic criteria.
